# Pharmacogenetics of Oral Antidiabetic Drugs

**DOI:** 10.1155/2013/686315

**Published:** 2013-11-13

**Authors:** Matthijs L. Becker, Ewan R. Pearson, Ivan Tkáč

**Affiliations:** ^1^Department of Epidemiology, Erasmus MC, 3015 CE Rotterdam, The Netherlands; ^2^Pharmacy Foundation of Haarlem Hospitals, 2035 RC Haarlem, The Netherlands; ^3^Medical Research Institute, University of Dundee, Dundee DD1 9SY, UK; ^4^Department of Internal Medicine 4, Faculty of Medicine, P. J. Šafárik University, 041 80 Košice, Slovakia; ^5^Department of Internal Medicine 4, L. Pasteur University Hospital, Rastislavova 43, 041 90 Košice, Slovakia

## Abstract

Oral antidiabetic drugs (OADs) are used for more than a half-century in the treatment of type 2 diabetes. Only in the last five years, intensive research has been conducted in the pharmacogenetics of these drugs based mainly on the retrospective register studies, but only a handful of associations detected in these studies were replicated. The gene variants in *CYP2C9*, *ABCC8/KCNJ11*, and *TCF7L2* were associated with the effect of sulfonylureas. *CYP2C9* encodes sulfonylurea metabolizing cytochrome P450 isoenzyme 2C9, *ABCC8* and *KCNJ11* genes encode proteins constituting ATP-sensitive K^+^ channel which is a therapeutic target for sulfonylureas, and *TCF7L2* is a gene with the strongest association with type 2 diabetes. *SLC22A1*, *SLC47A1*, and *ATM* gene variants were repeatedly associated with the response to metformin. *SLC22A1* and *SLC47A1* encode metformin transporters OCT1 and MATE1, respectively. The function of a gene variant near *ATM* gene identified by a genome-wide association study is not elucidated so far. The first variant associated with the response to gliptins is a polymorphism in the proximity of *CTRB1/2* gene which encodes chymotrypsinogen. Establishment of diabetes pharmacogenetics consortia and reduction in costs of genomics might lead to some significant clinical breakthroughs in this field in a near future.

## 1. Introduction

Type 2 diabetes (T2D) affects more than 5% of population of the developed countries and its prevalence increases worldwide [1]. The majority of patients with T2D start their treatment with oral antidiabetic drugs that influence two basic pathogenetic mechanisms in the development of T2D—insulin resistance or defects of insulin secretion. Beside glucose stimulated insulin secretion, more attention in the recent years is devoted to incretin augmented insulin secretion and new drugs were introduced in clinical practice which enhance the actions of incretins.

There is a considerable variability in the effect of antidiabetic drugs. This variability is caused by nonbiological and biological factors. Among nonbiological factors, psychological and social factors play an important role. These include compliance to medication, variable access to health care, and physician prescribing practices which are dependent on both international and national guidelines. 

Biological factors are related to pharmacokinetics and pharmacodynamics of drugs. Biological factors might be nongenetic, such as the influence of intestinal, hepatic and renal functions, or drug interactions predominantly on pharmacokinetics of drugs. Pharmacogenetics focuses on the study of genetic factors which influence the effect and side effects of different drugs with the final aim of personalizing the treatment of T2D. While clear implications for clinical practice based on pharmacogenetic knowledge exist only for some forms of monogenic diabetes [2], this review will focus on the more recent work undertaken to elucidate pharmacogenetic mechanism in common type 2 diabetes.

## 2. Methodological Aspects of Pharmacogenetic Studies in T2D

The optimal design of pharmacogenetic studies requires attention to some key methodological issues. Whilst the ideal study is a prospective genotype-blind study design with adequate statistical power, these are very costly, time-consuming and often require participation of multiple centers. Therefore, the majority of studies published used retrospective data retrieved from registers and databases. In the retrospective studies, it is possible to achieve reasonable sample sizes for pharmacogenetic studies, but it can be difficult to adjust for all confounding variables as these may not have been measured. However, as most confounding should not be genotype dependent, this should not be a major issue. 

 With respect to the outcomes used in pharmacogenetic studies, there are two main types of endpoints. Pathophysiological endpoints reflect the effect of gene variants on insulin resistance or insulin secretion. Such studies may yield novel knowledge on the role of different gene variants in the pathogenesis of T2D, but their clinical applicability might be limited. Thus, from the point of view of possible clinical implications, endpoints such as reduction of HbA1c, fasting plasma glucose, and postprandial plasma glucose are of the highest importance. Among them, both an achievement of therapeutic target of HbA1c<7% defined in guidelines and a reduction in HbA1c seem to be the most appropriate endpoints for pharmacogenetic studies in T2D [3].

 Different factors may confound the relationship between the studied gene variants and endpoints. When using achievement of HbA1c<7% or reduction in HbA1c as an endpoint, the strongest confounder is the baseline level of HbA1c, as it was shown in both the meta-analysis of clinical trials and also in the individual pharmacogenetic studies [4, 5]. That mean—if there is an imbalance in baseline HbA1c among the genotypes—one could expect higher response in the patients with higher baseline HbA1c values. On the other hand, higher baseline HbA1c might also reflect the effect of the gene variant on previous diabetes control. Thus, it is correct to show rough (unadjusted) data along with the models adjusted for confounding variables. It is also important to take into account whether the drug of interest is being used early on in the disease process, or as add-on therapy later in the disease, where there is less likely to be a large therapeutic effect. Covariates that are usually taken into account such as age, gender, BMI, and diabetes duration, are rarely significant predictors of the drug effect in pharmacogenetic studies. If the drug dose during the study is not constant, it is reasonable to adjust for drug dosage. Since the majority of the oral antidiabetic drugs are eliminated by the kidney, adjustment for a measure of renal function such as GFR or creatinine level is also frequently needed. Furthermore, if retrievable from databases, medication adherence and period of drug use are useful covariates.

We selected studies discussed in this review taking into account the above mentioned methodological aspects of pharmacogenetic studies in patients with T2D. We performed a search in the MEDLINE and Web of Knowledge databases using “pharmacogenetics OR pharmacogenomics” keyword in combinations with the names of groups of antidiabetic drugs or generic names of individual antidiabetic drugs. In the present review, we will discuss the mechanisms of associations between gene variants and the drug effect in the healthy subjects, in cell lines and in the patients with T2D. The studies which examined the effect of gene variants on the indices of glucose control will be presented only when at least 100 patients with T2D are included in the study.

## 3. Pharmacogenetics of Insulin Secretagogues 

Insulin secretagogues stimulate secretion of insulin from the pancreatic *β*-cells. Sulfonylureas have been used for more than a half-century, while nonsulfonylurea secretagogues meglitinides (glinides) have been used for approximately the last 15 years in the treatment of T2D. The most common side effects of both groups of drugs are hypoglycemia and weight gain. While previously sulfonylureas were considered as first-line treatment in less obese patients with T2D, more recent guidelines recommend both groups of drugs mainly in combination therapy with metformin as a second-line treatment [6]. 

### 3.1. Gene Variants Related to Pharmacokinetics of Insulin Secretagogues

#### 3.1.1. Cytochrome P450 Isoenzyme 2C9

Sulfonylureas are metabolized in the liver primarily by cytochrome P450 isoenzyme 2C9 encoded by *CYP*2*C*9 [7–10]. The major allele of this gene is *CYP*2*C*9*1. Two common nonsynonymous variants Arg144Cys (*CYP*2*C*9*2) and Ile359Leu (*CYP*2*C*9*3) were identified [7]. Studies on healthy subjects showed that the variant alleles are associated with increased plasma concentration and decreased clearance of sulfonylureas after oral administration [7, 10]. 

 The first robust study to confirm association of *CYP*2*C*9  variants with therapeutic response to sulfonylureas was Genetics of Diabetes Audit and Research Tayside Study (GoDARTS) which included 1073 patients treated with sulfonylureas. The 6% of the population who were carriers of two loss-of-function alleles (*2/*2 or *2/*3 or *3/*3) had a 0.5% greater reduction in HbA1c compared with wild-type homozygotes (*P* = 0.003) and were 3.4 times more likely to achieve on-treatment HbA1c<7% (*P* = 0.009) [5]. 

Further studies in patients with type 2 diabetes used indirect endpoints of sulfonylurea effect such as prescribed dose of sulfonylurea. Becker et al. analyzed 475 elderly patients included in the Rotterdam study who started sulfonylurea treatment. In the largest subgroup of patients treated with tolbutamide (*n* = 172), there was a significant difference in daily prescribed dose observed between genotypes. While the prescribed dose of tolbutamide increased in the carriers of *1/*1, *1/*2, or *2/*2 genotypes, there was very little tolbutamide dose increase in patients with *1/*3 or *2/*3 genotypes (*P* = 0.009) [11]. In a similar performed analysis from a different Dutch group which analyzed data from 207 incident sulfonylurea users, a trend towards lower stable glimepiride dose (*P* = 0.07) was observed in the carriers of the *CYP*2*C*9*3 allele [12]. 

Studies in healthy volunteers showed that variants in *CYP2C9*  influence also pharmacokinetics of nonsulfonylurea secretagogue nateglinide [13], while variant in *CYP2C8* is associated with pharmacokinetics of repaglinide [14]. Pharmacokinetics of both of these glinide drugs is also influenced by variant 521T>C in *SLCO1B1*, the gene encoding organic anionic transporter B1 (OATPB1) [15]. 

### 3.2. Gene Variants Related Pharmacodynamics of Insulin Secretagogues

The ATP-sensitive potassium (K_ATP_) channel plays crucial role in glucose stimulated insulin secretion. In physiologic condition, ATP produced by glucose oxidation in mitochondria leads to closure of K_ATP_ channel with subsequent depolarization of *β*-cell membrane, increased influx of calcium ions, and subsequent release of presynthesized insulin from the *β*-cell. Insulin secretagogues (both sulfonylureas and glinides) act by inducing K_ATP_ channel closure by binding on its constituting proteins. The inner pore of the K_ATP_ channel is constituted by four molecules of potassium inward rectifier 6.2 (Kir6.2) while the outside part is created by four molecules of sulfonylurea receptor 1 (SUR1) [16]. 

 Two sulfonylurea binding sites were identified on the K_ATP_ channel. The A-site is exclusively part of the SUR1 subunit whereas the B-site resides both on SUR1 and Kir6.2 subunits [17]. Sulfonylurea derivatives, chlorpropamide, tolbutamide, and gliclazide, as well as nateglinide and mitiglinide, bind exclusively to the A-site. Repaglinide binds exclusively to the B-site, while glibenclamide, glipizide, and glimepiride are AB-site binding drugs [18, 19]. 

 Mutations in genes encoding K_ATP_ channel proteins—*KCNJ11*  (encoding Kir6.2) and *ABCC8* (encoding SUR1)—lead to neonatal diabetes mellitus. Breakthrough pharmacogenetic studies showed that sulfonylureas are able to correct the defect caused by *KCNJ11* and *ABCC8* mutations resulting in patients (believed previously to have type 1 diabetes) being able to transition from long-term insulin therapy to sulfonylurea treatment [20, 21]. Thus, common variants in *KCNJ11* and *ABCC8* were logical selection also for pharmacogenetic studies in type 2 diabetes. 

#### 3.2.1. Kir6.2 and SUR1

Nonsynonymous common variants E23K in *KCNJ11* and S1369A in *ABCC8* were mostly studied in pharmacogenetic studies with insulin secretagogues. These two variants are in strong linkage disequilibrium so that any association signal from these two polymorphisms is genetically indistinguishable [22]. 

The most robust study which showed an association of *ABCC8* S1369A polymorphism with glycemic control was done in Chinese population. The study included 661 patients who were genotyped for 25 single nucleotide polymorphisms. *KCNJ11* rs5210 (different from E23K) and *ABCC8* S1369A polymorphisms were significantly associated with decrease in FPG. Association analysis of *ABCC8* S1369A with sulfonylurea response was replicated in an independent cohort of 607 patients. In the combined analysis of both cohorts, subjects with a *ABCC8* AA genotype had a significantly greater decrease in FPG (*P* < 0.001) and a 2-hour plasma glucose (*P* < 0.003), but only a borderline decrease in HbA1c (1.7 versus 1.4%, *P* = 0.06), in comparison with patients with SS genotype [23]. Lack of significance in HbA1c level reduction can be explained by relatively short 8-week duration of study, during which full effect of treatment on reduction in HbA1c level was not observed. Similar association was found by another Chinese group which evaluated glycemic response to 8-week gliclazide treatment. In a group of 115 patients with T2D, a greater reduction in HbA1c in response to 8-week gliclazide treatment in the carriers of the A-allele (SA and AA) compared to homozygous carriers of the S-allele (1.60 ± 1.39 versus 0.76 ± 1.70%, *P* = 0.044) was observed after adjustment for baseline HbA1c [24].

In Caucasian population, the association between *KCNJ11*  E23K and sulfonylurea efficacy was observed in the study of Javorsky et al. which included 101 patients treated with sulfonylurea after metformin monotherapy failure. In the dominant model, the carriers of the K-allele had higher reduction in HbA1c after 6-month therapy in comparison with EE homozygotes (1.04 ± 0.10 versus 0.79 ± 0.12%, *P* = 0.036) [25]. 

Previously, two studies did not find such association in Caucasian population. In the United Kingdom Prospective Diabetes Study (UKPDS) population, a group of 363 patients primarily assigned to sulfonylurea treatment was analyzed. No significant relationship was found between two *KCNJ11* polymorphisms (E23K and L270V) and the response to sulfonylurea. The evaluated outcome was based on two measurements of FPG within the first year of treatment, but not on HbA1c measurements [26]. Since titration of sulfonylurea dose was carried out in the UKPDS, this may have confounded the response phenotype. Another study performed in a group of 525 Italian patients showed that the carriers of K-allele had significantly higher probability of secondary sulfonylurea failure. In that study, secondary sulfonylurea failure was defined as not achieving FPG<300 mg/dL (16.7 mmol/L) on-treatment with combination of sulfonylurea as the first-choice drug and metformin as an add-on drug [27]. Thus, this study reported the failure of the combination of sulfonylurea with metformin, rather than the failure of sulfonylurea treatment itself. 

 Another study in Chinese population examined the association between *KCNJ11*  E23K and therapeutic response to repaglinide. He et al. found in a group of 100 patients treated for 24 weeks with repaglinide that the decrease in HbA1c was higher in patients with EK and KK genotypes than in EE homozygotes (EE: 1.52 ± 1.03%, EK: 2.33 ± 1.53%, and KK: 2.65 ± 1.73%, *P* = 0.022) [28]. The authors did not adjust the decrease in HbA1c for the baseline levels; thus, it is not possible to exclude that this effect was driven by an effect of the E23K variant on baseline HbA1c.

Clinical studies showed higher efficacy of sulfonylurea treatment in risk allele carriers suggesting that, in line with the studies in neonatal diabetes, sulfonylureas and possibly also glinides are able to correct the defect in insulin secretion by binding to the proteins of the K_ATP_ channel resulting in its closure. Studies in cell lines transfected by cloned channels with *KCNJ11  *K23-*ABCC8* A1369 haplotype shed some light on observed differences among different sulfonylureas and glinides to affect insulin secretion in carriers of risk haplotype. K23/A1369 carriers were more sensitive to inhibition of K_ATP_ channel by gliclazide and mitiglinide in comparison with E23/S1369 haplotype carriers. In contrast, E23/S1369 carriers were more sensitive to glimepiride, chlorpropamide, and tolbutamide, whilst there was no difference in K_ATP_ channel sensitivity between the two haplotypes for glibenclamide, glipizide, repaglinide, and nateglinide [18, 19].

#### 3.2.2. Transcription Factor 7-Like 2

Among more than 50 gene variants associated with T2D, the variants in transcription factor 7-like 2 (*TCF7L2*) gene are the strongest predictors of increased risk of developing type 2 diabetes, a 40% increased risk per allele [29, 30]. TCF7L2 is a nuclear factor which binds *β*-catenin, mediates Wnt-signaling, relates to normal development of pancreas during embryogenesis, and affects secretion of glucagon-like peptide 1 (GLP-1) by L-cells in the small intestine [31]. Several studies observed that carriers of the risk T-allele of *TCF7L2* rs7903146 polymorphism have reduced insulin secretion [32, 33]. Mechanisms whereby altered TCF7L2 production and/or function may contribute to the development of type 2 diabetes are not fully understood but likely include a decrease in *β*-cell mass, impaired insulin processing or release, and impaired incretin signaling in *β*-cells [34, 35].

 GoDARTS was the first pharmacogenetic study to address the relationship between the *TCF7L2* rs12255372 (G/T) and rs7903146 (C/T) gene variants and response to sulphonylurea therapy in type 2 diabetic patients. In that study in 901 Scottish patients with type 2 diabetes, subjects with the rs12255372TT genotype had approximately two times higher probability for early sulphonylurea treatment failure (HbA1c>7% within the period of 3–12 months after starting sulphonylurea therapy) compared to those with the CC genotype. In a complementary approach, a linear regression model was used with minimal HbA1c during treatment within a year after sulfonylurea initiation as the dependent variable. The predicted HbA1c on-treatment was for rs1225372 GG genotype 7.0%, while for TT genotype, it was 7.33%. Similar results were observed for rs7903146 [36].

 Results of this study were replicated by two study groups. Javorský et al. found in a group of 101 Slovakian patients that the reduction in HbA1c after six months of sulphonylurea therapy (ΔHbA1c) was significantly lower in the CT+TT genotype group in comparison with the CC homozygotes for rs7903146 genotype. The absolute difference in ΔHbA1c between the two groups was 0.35% (*P* = 0.006) [37], that is, very similar to the value observed in GoDARTS study. In another study in a German population, Holstein et al. included 179 patients treated with sulfonylureas and analyzed sulfonylurea treatment failure after 6 months according to rs7903146 genotypes. They found more than twice the probability of sulfonylurea failure in TT homozygotes in comparison with CC homozygotes (OR 2.09; 95% CI 1.02–4.27, *P* = 0.043). In this study, the adjustment for the baseline HbA1c values was not performed [38].

## 4. Pharmacogenetics of Metformin

Metformin has been used for the treatment of type 2 diabetes mellitus since 1959 and is still the cornerstone in the treatment of this disease. At physiological pH, metformin is positively charged and therefore very hydrophilic, giving it some interesting pharmacokinetic properties [39]. First, metformin is not metabolized in the body but very efficiently excreted in the urine. Therefore, the glucose lowering effect of metformin is not affected by genetic variation in metabolizing enzymes. Second, metformin cannot diffuse through membranes passively, but it is dependent on drug transporters for the absorption, distribution, and elimination of metformin [39]. The initial pharmacogenetic research focused on the role of metformin transporters while the most extensive study thus far is the genome-wide association study (GWAS).

### 4.1. Genome-Wide Association Study

In GWAS, Zhou et al. genotyped more than 700,000 polymorphisms in 1,024 metformin users from the GoDARTs study [40]. They identified 14 polymorphisms in a locus containing the *ATM* gene that were associated with the ability to reach the treatment goal of an HbA1c<7%. The strongest association was with the rs11212617 polymorphism. Participants had a 1.64 times higher change of reaching the treatment goal for each minor allele with a *P* value of 1.9 × 10^−7^. The authors subsequently genotyped this polymorphism in two independent populations (another GoDARTS population and the UKPDS population) for replication. And in both populations, a significant association was found with treatment response. The combined effect was 1.35 times higher change of reaching the treatment goal (*P* = 2.9 × 10^−9^) for each minor allele. The secondary analysis was the association between this polymorphism and the reduction in HbA1c, and a 0.11% larger reduction in HbA1c per minor allele (*P* = 6.6 × 10^−7^) was found in the three cohorts combined. In the replication study by van Leeuwen et al., the rs11212617 polymorphism was studied in three independent populations, the Diabetes Care System (DCS) West-Friesland (*n* = 929), the Rotterdam Study (*n* = 182), and the CARDS Trial (*n* = 254). They used the same endpoint as was used in the initial GWAS by Zhou et al. In the three populations, the combined odds ratio was 1.24 with a *P* value of 0.016. For the secondary endpoint, the reduction in HbA1c per minor allele, no significant association was found [41].

The rs11212617 polymorphism has also been genotyped in the at-risk population of the aforementioned DPP trial. However, Florez et al. describe that no association was found between this polymorphism and the incidence of diabetes in the participants randomized to metformin therapy [42].

### 4.2. Genes Related to Pharmacokinetics of Metformin

#### 4.2.1. Organic Cation Transporters

Shu et al. were the first to study the effect of genetic variation in the *SLC22A1* gene encoding the OCT1 transporter and the glucose lowering effect of metformin both in animal model and in healthy volunteers. They identified four polymorphisms in the *SLC22A1* gene coding for a change in amino-acid sequence (R61C, G401S, 420del, or G465R) and studied the association with the glucose lowering effect. In the subjects carrying one or more variant alleles, the metformin plasma levels were higher and the glucose lowering effect during a glucose tolerance test was impaired [43, 44]. In Caucasians, R61C and 420del are the most important genetic variants, because the variant alleles occur frequently and decrease transporter activity [45]. For R61C, it has been shown that this variant strongly reduces OCT1 protein expression [46]. Tzvetkov et al. identified that OCT1 beside the liver is also expressed on the apical side of tubules and that healthy volunteers carrying 420del alleles in OCT1 had an increased renal metformin clearance, due to a decrease in reabsorbance [47].

 Strikingly, in the largest study in subjects with T2D performed by Zhou et al., no association was found [48]. In this study, 1,531 type 2 diabetes mellitus patients participating in the GoDARTS were included. Neither the R61C nor the 420del variant had a significant association with various endpoints, including the maximum HbA1c reduction in the 18 months after start of metformin therapy and the ability to reach a treatment target of HbA1c<7%. 

Christensen et al. studied the effect of eleven polymorphisms in various transporters including OCT1 on the through plasma levels and the glucose lowering effect in 151 diabetes mellitus patients in whom metformin was added to the previous insulin therapy. Patients with one or two variant alleles of 420del had lower trough plasma levels. In this study, two other polymorphisms, G401S and rs461473 in an intronic region not coding for an amino-acid change, were significantly associated with the glucose lowering effect after start with metformin therapy [49].

Two other polymorphisms that have been described in the OCT1 transporter are the rs622342 and the M408V polymorphisms. Becker et al. screened the *SLC22A1* gene using tagging polymorphisms and found that the rs622342 polymorphism was significantly associated with the glucose lowering effect in 102 incident metformin users [50]. This result has not been replicated thus far. The M408V variant has been associated with gastrointestinal side effects by Tarasova et al. in 246 metformin users [51]. However, Shu et al. reported that this genetic variation is not associated with reduction in metformin transporter activity [43]. 

In Asians, the frequencies of R61C and 420del variants are low and no polymorphisms in the *SLC22A1* gene have been described that occur frequently and have a reduced transporter activity [45]. Several studies have suggested that genetic variation in OCT2 has a more important role in Asians than OCT1. OCT2, encoded by the *SLC22A2* gene, is expressed in the basolateral membrane of the renal epithelial cells. In three studies in Asian populations, an association between A270S and renal metformin clearance or plasma lactate concentrations has been described [52–54]. An association between A270S and renal clearance was also found in a study performed in the USA [55]. 

#### 4.2.2. Multidrug and Toxin Extrusion Transporters

Metformin is also a substrate for the multidrug and toxin extrusion 1 (MATE1) transporter. This transporter, encoded by the *SLC47A1* gene, is strongly expressed in the brush border membrane of the kidney and the bile canaliculi in the liver. It is believed to facilitate the excretion of compounds such as metformin in the urine and bile. Becker et al. were the first to identify that the tagging polymorphism rs2289669 (G/A) was associated with the HbA1c lowering effect in incident metformin users [56]. They subsequently described an interaction with the rs622342 polymorphism in the *SLC22A1* gene, identified earlier by this group [57]. Jablonski et al. studied the effect of genetic variation in 40 candidate genes in 2,994 participants of the Diabetes Prevention Program (DPP) study that were at risk for developing diabetes mellitus [58]. They randomized the participants to placebo, metformin, or lifestyle intervention and observed the finding that the rs8065082 polymorphism is associated with metformin response in the at-risk population treated with metformin. Since this polymorphism is in tight linkage disequilibrium with rs2289669 (*r* ^2^ ≈ 0, 8) and the effect was consistent in both studies, this is considered as a replication of the previous findings of Becker et al. For the other polymorphisms, no significant associations were found after correction for multiple testing. 

Also Tkáč et al. identified in 148 incident metformin users that the rs2289669 polymorphism is associated with HbA1c reduction. The homozygous carriers of the minor A allele had twofold reduction in HbA1c in comparison with the G-allele carriers (1.10 ± 0.18% versus 0.55 ± 0.09%) [59]. Stocker et al. studied the g.−66T>C polymorphism in the promoter region of the *SLC47A1* gene and found that in healthy volunteers, the variant allele resulted in lower glucose levels, and in 145 incident metformin users, it resulted in an increased HbA1c lowering effect [60]. In a subsequent study, they identified that this polymorphism was not associated with metformin disposition and that the effect of the g.−66T>C polymorphism was larger in patients with normal functioning OCT1 alleles [60]. Most likely, the variant allele associated with reduced expression of the *SLC47A1* gene results in higher hepatic plasma levels, due to reduced efflux transporter activity. In patients with a reduced OCT1 influx transporter activity, the hepatic plasma levels will be lower, masking the effect of the *SLC47A1* efflux polymorphism.

Also the MATE2 transporter, encoded by the *SLC47A2* gene, is expressed at the brush border membrane of the kidney. This transporter has two functional isoforms, MATE2 and MATE2-K. The g.−130G>A polymorphism is situated in the basal promoter region of MATE2-K and results in an increase in promoter activity. In the study by Choi et al., this polymorphism was associated with the HbA1c lowering effect in 248 incident metformin users [61]. In the study by Stocker et al., the renal clearance of metformin was diminished in healthy volunteers carrying a variant allele and the glucose lowering effect increased [60]. The effect of the g.−130G>A polymorphism was influenced by the previously described g.−66T>C polymorphism in the *SLC47A1* gene. Both MATE1 and MATE2 are coexpressed at the apical membrane of the kidney, possibly explaining their mutual effect [60].

## 5. Pharmacogenetics of Thiazolidinediones

The group of thiazolidinediones, including troglitazone, pioglitazone, and rosiglitazone, were introduced in the late 1990s. These drugs are agonists of the peroxisome proliferator-activated receptor (PPAR-*γ*). Activation of this receptor regulates the transcription of hundreds of genes, involved in lipid and glucose metabolism. Effects associated with PPAR-*γ* activation include decreased insulin resistance, decreased leptin levels, and increased adiponectin levels. Troglitazone has been withdrawn from the market worldwide due to liver injury and rosiglitazone has been withdrawn from the market in Europe due to an increased risk of cardiovascular events and put under restrictions in the USA.

### 5.1. Genes Related to Pharmacokinetics of Thiazolidinediones

#### 5.1.1. Cytochrome P450 2C8

Both pioglitazone and rosiglitazone are metabolized by the cytochrome P450 2C8 isoenzyme. The CYP2C8*3 and *11 polymorphisms, coding for a reduced functioning CYP2C8 enzyme, have been studied in relation to the pharmacokinetics of these drugs. Three studies associated these polymorphisms with increased pioglitazone and rosiglitazone plasma levels [62–64] and one study did not find such as association [65]. All four studies were performed in healthy volunteers. Only in the study including patients with type 2 diabetes treated by rosiglitazone from the South Danish Diabetes Study cohort (*n* = 187), the association between *CYP*2*C*8*3 variant and change HbA1c was studied. The authors found reduced therapeutic response and a lower risk for developing edemas in *CYP*2*C*8*3 carriers. In this study rosiglitazone was added to the previous insulin treatment and it was allowed to change the insulin dose during the treatment which could have influenced the observed study results [65]. 

### 5.2. Genes Related to Pharmacodynamics of Thiazolidinediones

#### 5.2.1. PPAR-*γ*

The obvious first choice in the pharmacogenetic research after thiazolidinediones was the *PPAR-*γ** gene. One polymorphism in this gene, the P12A polymorphism (rs1801282), was identified in 1997 and studied extensively in the relation to the incidence of type 2 diabetes mellitus [66]. In a meta-analysis published in 2010, 66 studies were included and the risk of developing type 2 diabetes mellitus was 14 percent lower for each minor allele of this polymorphism [67]. Three studies examined the association of this polymorphism with the glucose lowering response of thiazolidinediones. In the study by Hsieh et al. in 250 diabetes patients and the study by Kang et al. in 198 diabetes patients, the alanine (A) allele was associated with a stronger reduction in HbA1c and fasting plasma glucose levels in pioglitazone and rosiglitazone users, respectively [68, 69]. On the contrary, other studies did not find an association between this polymorphism and thiazolidinedione therapy. In the largest study thus far by Florez et al., no association was found with insulin sensitivity in 340 participants of the DPP study using troglitazone [70]. Blüher et al. (131 pioglitazone users) and Namvaran et al. (101 pioglitazone users) did not find an association between this polymorphism and HbA1c reduction, insulin sensitivity, and therapeutic response, respectively [71, 72]. Whether there is a true association between the P12A polymorphism and response to thiazolidinedione therapy remains to be determined.

#### 5.2.2. PGC-1*α*

The proliferator-activated receptor-*γ* coactivator-1*α* (PGC-1*α*) is a regulator of PPAR-*γ*, and it is associated with the risk of developing diabetes. Both the T394T and the G482S variants in this gene were related to response to rosiglitazone therapy in the study by Kang et al. in 241 patients [69]. But in the study by Hsieh et al., no association was found with the G482S polymorphism in 250 pioglitazone users [68].

#### 5.2.3. Adiponectin

The binding of thiazolidinediones to PPAR-*γ* regulates numerous other genes, and some of these genes have been subject of pharmacogenetic research. Adiponectin is the best studied gene that is regulated by PPAR-*γ*. Three polymorphisms in this gene, − 11377C>G, +45T>G, and +276G>T, were previously associated with obesity development and the incidence of type 2 diabetes mellitus, and the effect of these polymorphisms on thiazolidinedione response was studied. In the study by Eun et al., the +45T>G and +276G>T polymorphisms were associated with the change in HbA1c levels in 166 rosiglitazone users [73]. The association with the −11377C>G polymorphism was found in the study by Li et al. They found an association with the change in HbA1c level after start of pioglitazone treatment in 113 users, but no association was found with the +45T>G polymorphism [74]. In the study by Namvaran et al., no association was found between the +45T>G polymorphism and response to pioglitazone in 101 users either [75]. 

## 6. Pharmacogenetics of Incretin Mimetics

The incretin effect is mediated by stimulation of insulin secretion from pancreatic *β*-cells after ingestion of glucose or mixed meals by intestinal hormones glucagon-like peptide-1 (GLP-1) and glucose-dependent insulinotropic peptide (GIP) [76]. In a pilot study including 88 healthy individuals, it was shown that two GLP-1 receptor (*GLP1R*) variants were associated with altered *β*-cell sensitivity to GLP-1 infusion [77]. Variation in the GIP receptor (*GIPR*) locus was reported to influence the responses of the glucose and insulin to an oral glucose challenge in a meta-analysis of nine GWAS which included 15,234 nondiabetic subjects [78]. 

 Besides the gene variants encoding GLP-1 and GIP receptors, several T2D related gene variants were reported to be associated with the incretin effect. Gene variants in *TCF7L2* [79–81], voltage-gated potassium channel, KQT-like subfamily, member 1 (*KCNQ1*) [82], and wolframin 1 (*WFS1*) [83] were associated with decreased incretin secretion, decreased sensitivity of GLP-1 or GIP receptors, or with decreased suppression of glucagon secretion [84].

 Since endogenous and exogenously applied GLP-1 is rapidly degraded by dipeptidylpeptidase-4, two pharmacologic approaches were developed to bypass this limitation and to enhance the incretin effect: GLP-1 receptor antagonists are resistant to DPP-4 and applied subcutaneously. Another possibility is to inhibit DPP-4 by orally applied DPP-4 inhibitors (gliptins) [76].

### 6.1. Pharmacogenetics of Gliptins

#### 6.1.1. Chymotrypsinogen

Until recently, no pharmacogenetic study with incretin mimetics was published. In their recently published study, ‘t Haart et al. used Metabochip (including tests for approximately 186,000 SNPs that previously were associated with metabolic or cardiovascular disease) and showed that three genetic loci (*TMEM114, CHST3,* and *CTRB1/2*) had large effects on GLP-1 stimulated insulin secretion during hyperinsulinemic clamp [85]. A pharmacogenetic study in two independent populations—Dutch DCS West Friesland and GoDARTS—was performed. This study included 173 patients treated by GLP-1 receptor antagonists, in which no association of glycemic control after treatment with any of the three gene variants was observed. Among 354 patients treated with gliptins, there was a significant association between the rs7202877 T/G variant in the proximity to *CTRB1/2* gene encoding chymotrypsinogen and the response to gliptin treatment. 10% patients carrying the minor G-allele showed a significantly smaller decrease in HbA1c (in average by 0.5%) in comparison to TT homozygotes [85].

## 7. Conclusions

There are a few areas where genetics is used to guide therapy. This is becoming mainstream in the field of cancer therapy where somatic mutations can determine the choice of treatment. Out with the cancer setting, there are currently limited examples of clinical utility of genetics, with probably the most accepted use being HLA genotyping prior to commencing the antiretroviral, abacavir. The use of genotyping abolishes hypersensitivity reactions to the drug [86]. Diabetes is one of the few other disease areas where genotype is used to guide therapy, albeit in rare monogenic subtypes [20]. In this review, we have not focused on these rare subtypes, but addressed the evidence for the role of genetic variants in the pharmacogenetics of common type 2 diabetes. This field is progressing rapidly and with increasing rigor. The most recent and promising advances have been in the field of metformin therapy. Whilst at present, there is no convincing clinical role for genotype led prescribing, some of the organic cation transporter variants do offer such promise, and the evidence is starting to accumulate to sufficient level to justify a genotype led clinical trial. We should recognize that pharmacogenetics can also be a useful tool to understand drug mechanism, and this is highlighted by the recent genome-wide association study that points to a novel biological mechanism of action of metformin. With increased collaboration between groups, establishment of diabetes pharmacogenetics consortia, and with reduction in costs of genomics, we anticipate that the next five years should lead to some significant clinical breakthroughs in this field.
